# Comparison of methods for miRNA isolation and quantification from ovine plasma

**DOI:** 10.1038/s41598-020-57659-7

**Published:** 2020-01-21

**Authors:** Kathryn Wright, Kumudika de Silva, Auriol C. Purdie, Karren M. Plain

**Affiliations:** The University of Sydney, Faculty of Science, Sydney School of Veterinary Science, Sydney, Australia

**Keywords:** High-throughput screening, miRNAs

## Abstract

microRNA (miRNA) are promising candidates for disease biomarkers as they are abundant in circulation, highly stable in biological fluids and may yield diagnostic biomarker signatures. The reported issues with miRNA isolation using traditional RNA reagents necessitates the optimisation of miRNA isolation from challenging samples. In this study we compared six commercial RNA extraction kits to evaluate their ability to isolate miRNA from ovine plasma. We also compared three methods for quantification of small RNA extracted from plasma to determine the most reliable. Using minimal sample inputs of fresh and frozen plasma from five sheep, we compared the six kits (Kit A-F) using quantitative PCR. Operational factors were also assessed for each kit. Kits A and B provided the best detection of the miRNA qPCR reference genes across fresh and frozen samples (p < 0.001) followed by Kit C. The Qubit and microRNA assay provided the least variation (% CV 5.47, SEM ± 0.07), followed by the NanoDrop (% CV 7.01, SEM ± 0.92) and Agilent Bioanalyzer (% CV 59.21, SEM ± 1.31). We identify Kit A to be optimal for isolating miRNA from small volumes of fresh and frozen ovine plasma, and Kit B the top performing kit taking into consideration miRNA detection and operational factors. The Qubit fluorometer using a microRNA assay was the most reliable miRNA quantification method.

## Introduction

At the forefront of disease research in both human and veterinary medicine, predictive and diagnostic biomarkers have the potential to be highly specific and informative. Biofluids such as plasma, serum, saliva, or urine are common targets for biomarker profiling as they are collected by minimally invasive techniques and contain biologically relevant molecules which may be indicative of disease outcomes^1^. One such biomarker that is frequently assessed is microRNA (miRNA), a small (approximately 22 nucleotides in length) subset of non-coding RNA which are critical mediators of gene expression. These short transcripts limit protein translation through a process known as “gene silencing”, where miRNA bind to mRNA molecules and either transiently block translation of proteins, or degrade the bound mRNA^2^.

The expression of miRNA is notably altered during both bacterial and viral infection, as well as diseases such as cancer and diabetes^3–6^. These molecules are thought to be excellent biomarker candidates as they are stable in blood and biofluids despite high levels of RNase, and are abundant in the circulation associated with extracellular vesicles and protein complexes^7^. In their role as regulators of translation, miRNA represent a complex interplay between the host immune system and invading pathogens, supporting the need for research into their biological function and control of disease processes.

Challenges remain with respect to the isolation and accurate quantification of miRNA, especially from complex samples such as plasma. The small size of miRNA, along with high levels of contaminating protein and inhibitors present in plasma, reduces the efficacy of current molecular isolation methods. Commercially available extraction kits are commonly used in place of traditional RNA isolation protocols as they offer a faster and often simpler method of RNA isolation: these kits may be further specialised for miRNA isolation and a range of sample types, including serum and plasma, plant material, and tissue. The association of miRNAs with protein complexes such as lipoproteins and RNA-binding proteins further complicates isolation and quantification, as co-isolation of proteins may confound quantitative measurements and interfere with downstream applications^8,9^. Sample quality and processing are important factors affecting the quality and quantity of miRNA isolated; the profile of commonly used reference miRNA can be significantly altered in haemolysed plasma samples, while rupturing of cellular components of blood may alter the miRNA profile^10–13^. Similarly, the configuration of the miRNA itself may influence its ability to be effectively profiled. Sequences with lower GC content and stable secondary duplex structures have been shown to be less stable and therefore at risk of being lost during the extraction process^14^. It is evident that a range of factors affect the success of miRNA isolation, and therefore it is important that the chosen extraction protocol can tolerate these sample constraints.

Often, utilising a combination of sample lysis and silica-based spin column technology, commercial kits precipitate and bind RNA to the column membrane, allowing for easy elution and reduced sample loss when sequential pelleting of the RNA is performed. While many kits cater for total RNA isolation, several newer options are specialised for miRNA isolation and provide protocols for the isolation of either total RNA including miRNA, or for the selective isolation of miRNA only. The wide range of kits currently on the market provides a surplus of options for RNA and miRNA research; however, as there are many options, it is often necessary to test several kits for their suitability for specific applications and minimisation of extraction bias based on GC content and thermostability.

The measurement and quantification of isolated miRNA are similarly important aspects. Traditionally, spectrophotometric methods have been used for RNA measurement that rely on the absorbance of UV-visible light at specific wavelengths to measure particle concentration, however at low concentrations this technique can often present inaccurate and inflated results^15^. Methods relying on fluorometric detection of nucleic acids may be more sensitive alternatives with greater tolerance for the presence of contaminants.

To date there is limited knowledge and research into the plasma miRNome of sheep, and there are currently no reports on the optimal methods for ovine miRNA analysis. While miRNA research in human or murine models may provide the groundwork for ruminant studies, the biological and physiological differences in plasma composition and the variability of plasma component levels due to diet and metabolism necessitates investigation of the applicability of these kits and protocols for ruminant samples^16^. Thus, an evaluation of the suitability of isolation protocols for specific sample types was undertaken. Discovery and profiling of biomarkers, as one of the main uses of isolated miRNA, necessitates that the starting volume is kept minimal, while still maintaining the sensitivity to detect low abundance targets. Here we have compared the suitability of six commercial kits, some specialised for miRNA isolation and others more broadly for several RNA populations, for applicability to the isolation of miRNA from low volumes ovine plasma. Several miRNAs, with varying GC content and thermostability (calculated using Mfold^17^), were quantified by qPCR and common RNA quantification methods were compared.

## Results

**Figure 1:**
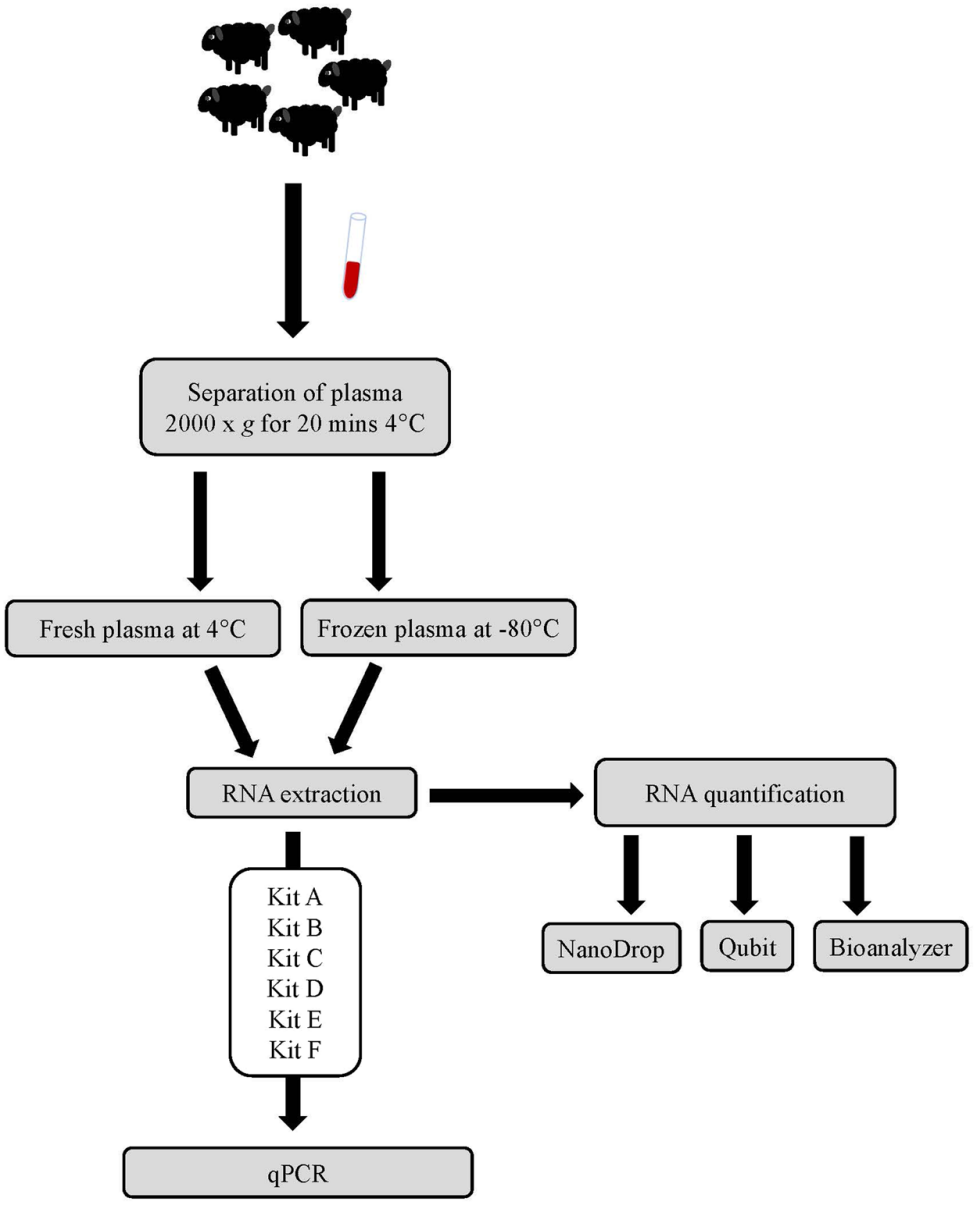
Overview of experimental design. Whole blood was collected from 5 healthy sheep and plasma stored at either −80 °C or used fresh. RNA was extracted from both the fresh and frozen plasma using the 6 isolation kits and compared using qPCR. RNA extracted from frozen plasma samples was also quantified using 3 different methods to compare the most appropriate method for measuring quantity and quality.

A comparison of six different commercial kits for miRNA isolation from fresh or frozen ovine plasma was performed (Kits A-F). Additionally, three methods of RNA quantification were compared. An overview of the experimental design used for the assessment of isolation methods for ovine plasma miRNA is shown in Fig. 1.

### RNA quantification

**Figure 2:**
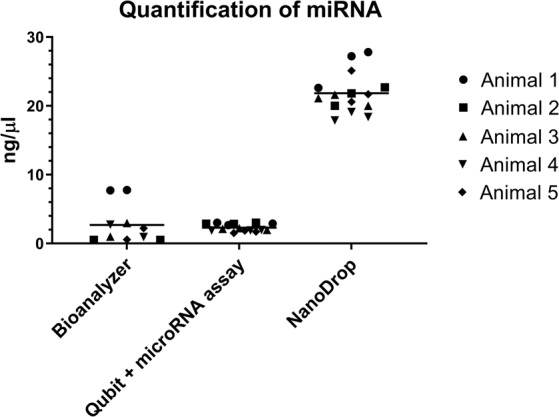
Comparison of miRNA quantification methods. RNA extracts from 5 frozen ovine plasma samples were quantified using 3 different methodologies. Multiple measurements were taken for each sample, with the mean for each method indicated by a horizontal line.

All three methods of RNA quantification assessed were able to detect RNA in extracts from frozen plasma. The Qubit microRNA assay was able to quantify low abundance targets with the least within-sample variation (average % CV 5.47, SEM ± 0.07), while also detecting RNA from each individual sample (Fig. 2). Both the NanoDrop and the Bioanalyzer failed to detect any nucleic acid in more than one replicate extraction. The Bioanalyzer and the Qubit assay produced similar measurements of samples, whereas the NanoDrop returned considerably higher values. Both the Bioanalyzer and NanoDrop had greater variation between readings (average % CV 59.21, SEM ± 1.31 and % CV 7.01, SEM ± 0.92 respectively) compared to the Qubit.

### Detection of miRNA by qPCR

**Figure 3:**
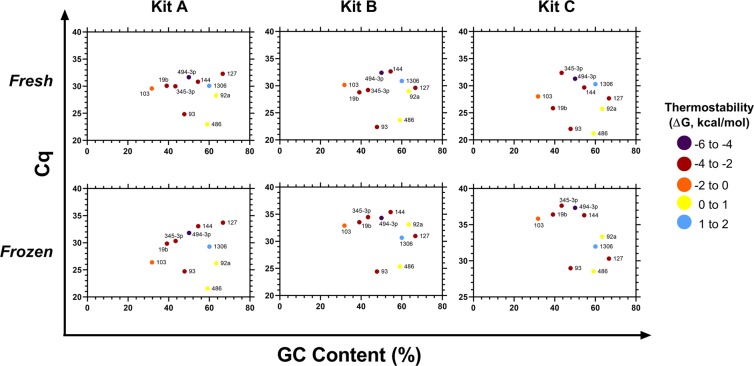
Performance of commercial RNA extraction kits A-C. For each kit (**A**–**C**) and treatment, Cq values for each of the 10 miRNA were plotted against the corresponding GC % and colour coded based on the thermostability of their secondary structures (ΔG, kcal/mol). Secondary structures and ΔG were calculated using Mfold.

**Figure 4:**
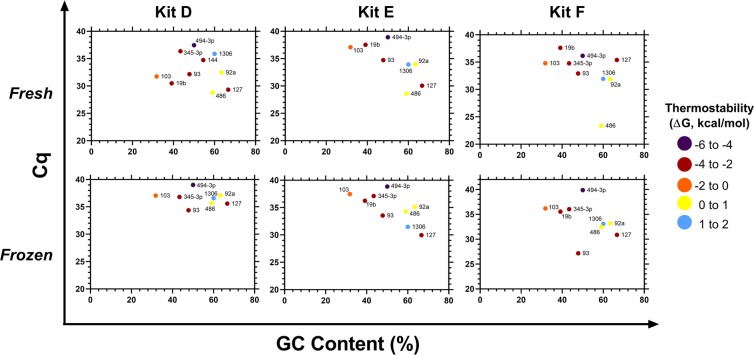
Performance of commercial RNA extraction kits D-F. For each kit (**D**–**F**) and treatment, Cq values for each of the 10 miRNA were plotted against the corresponding GC % and colour coded based on the thermostability of their secondary structures (ΔG, kcal/mol). Secondary structures and ΔG were calculated using Mfold. Kit D failed to detect miRs -144 and 19b in frozen samples. Kit E failed to detect miRs -144 and -345-3p in fresh samples, and miR-144 in frozen samples. Kit F failed to detect miR-144 in both fresh and frozen samples.

**Figure 5:**
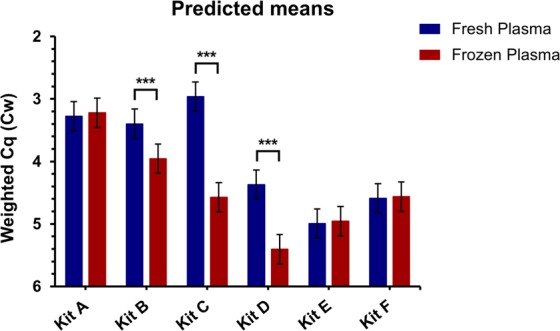
Overall kit performance between fresh and frozen samples. REML predicted means (±SEM) of kit performance across both fresh and frozen samples. Means represent the average Cw of 5 biological and 2 technical replicates (n = 10) for Kits A-C & E, and 5 biological and 1 technical replicate (n = 5) for Kits D & F. Significant differences between fresh and frozen samples (p < 0.001) are indicated by an asterisk.

To assess the success of miRNA extractions using different commercial kits, ten miRNA were detected by qPCR from each of the sample extracts (Figs. 3 and 4). Overall, across the 10 miRNA (considering the impact of varying GC content) using fresh and frozen samples, Kits A and B outperformed Kits D, E, and F (p < 0.001). For both fresh and frozen plasma, Kit A was the best overall, with Kits B, C and D having inferior performance on frozen samples (p < 0.001). While Kit C outperformed both Kits A and B for fresh plasma samples, for frozen samples its performance was significantly worse (p < 0.001). There was no significant difference between fresh and frozen samples for Kits E and F, however they had much higher Cq (quantification cycle) values across multiple miRNAs than the top performing kits (Figs. 3–5). Kit D failed to detect miR-144 and −19b in frozen samples. For Kit E miR-144 was not detected in fresh or frozen samples, and miR-345-3p was not detected in fresh samples. Similarly, Kit F failed to detect miR-144 in either fresh or frozen plasma.

The repeatability of extractions from each kit was also assessed, with the coefficient of variation (CV) calculated for both fresh and frozen samples across all miRNA. CV was calculated for each of the five biological replicates to provide an overview of the variability between technical replicates (Supplementary Figure 1). Kits A, B and F showed the greatest repeatability between technical replicates, whereas Kits C, D and E displayed larger variation both across replicates and between fresh and frozen samples.

The amount of cDNA input for each sample was calculated and analysed using a two-way ANOVA. There was no significant difference in the cDNA quantity across kits (p = 0.934). There was a significant difference between the cDNA input of fresh and frozen plasma in Kit B, with fresh samples having a higher amount of cDNA in the qPCR reaction on average compared to the frozen plasma samples (p < 0.001).

### Comparison of operational factors between different miRNA isolation methods

A range of operational factors were also considered, consisting of subjective aspects associated with the use and application of each kit for miRNA isolation (Table 1). These factors included RNA yield, ease of use, time input, sample volume input, and cost per sample for each method.

**Table 1:** Comparison of commercial miRNA isolation kits based on operational factors.

EXTRACTION KIT	EASE OF USE	TIME INPUT (APPROX)	SAMPLE INPUT	COST (PER SAMPLE) AUD $
Kit A	+++	+ (45 minutes)	++ (200 μl)	+++ ($20.88)
Kit B	+++	+ (40 minutes)	++ (200 μl)	++ ($15.52)
Kit C	+	+++ (2.5 hours)	++ (200 μl)	++ ($14.32)
Kit D	+	+ (40 minutes)	+ (100 μl)	++ ($13.40)
Kit E	++	+ (25 minutes)	++ (200 μl)	++ ($19.20)
Kit F	++	+ (25 minutes)	++ (200 μl)	+ ($7.08)
**BEST IN CATEGORY**	A/B	A/B/D/E/F	D	F
**BEST OVERALL: KIT A + B**	+ = low, ++ = medium, +++ = high			

**Figure 6:**
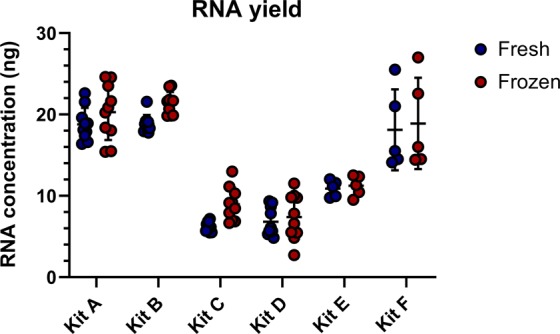
RNA yield from commercial extraction kits. The concentration of RNA extracted from each of the kits was measured using the Qubit fluorometer and RNA HS assay kit. Values represent the total amount of RNA extracted from each 200 or 100 μl volume of plasma. Elution volumes are: Kit A (12 μl), Kit B (18 μl), Kit C (13 μl), Kit D (50 μl), Kit E (30 μl), Kit F (50 μl). Each data point is a single replicate (n = 10 for kits A-C & E, and n = 5 for Kits D & F). Error bars indicate standard deviation.

The average yield from each of the kits was considered as one of the key factors (Fig. 6). Kits A and B yielded the highest amount of RNA, with Kit C also performing well with fresh plasma. For the kits with the highest RNA yield with both fresh and frozen plasma, between 18–20 ng total was extracted from 200 µl of plasma. Kits with larger elution volumes such Kit F (50 µl) had comparable yields, however, were less concentrated than the kits with lower elution volumes, like Kit A (12 µl). Kit D had the lowest minimum sample input of 100 µl of plasma compared to 200 µl in the other extraction kits, while several kits (A-C) provided a higher maximum input volume of up to 600 µl to increase processing volume. Regarding the ease of use and time required per extraction, Kits A and B were the top performing methods, requiring the least time input (approximately 40–45 minutes in our hands). Conversely, Kits C and D performed the worst, with either the need for extra equipment or low usability of kit components. Kits A and B performed best across yield and ease of use and were also among the best for time input and sample input along with Kits D, E and F. While Kit A was the most expensive, Kit B was in the mid-range and had the best performance overall across the operational categories. Although Kit D and Kit F were the most affordable choices, they also delivered the lowest miRNA yield from ovine plasma in our hands.

Similar operational factors were also considered for the methods of RNA quantification (Table 2); the factors assessed were the same as for the commercial miRNA isolation kits with the exception of yield, which was replaced by ‘high-throughput’, i.e. the ability to process many samples in large batches. The NanoDrop performed best across all operational factors, with the Qubit and microRNA assay the second best, followed by the Agilent Bioanalyzer.

**Table 2:** Comparison of three common quantification methods for miRNA-containing RNA.

QUANTIFICATION METHOD	EASE OF USE	TIME INPUT	SAMPLE INPUT	COST (PER ASSAY)	HIGH THROUGHPUT
Agilent Bioanalyzer + small RNA reagents (A)	+	+++	+	+++	+
Qubit fluorometer + microRNA Assay (B)	++	++	+	++	++
NanoDrop Spectrophotometer (C)	+++	+	+	+	+++
**BEST IN CATEGORY**	C	C	A/B/C	C	C
**BEST OVERALL: METHOD C**	+ = low, ++ = medium, +++ = high				

## Discussion

Optimisation of the nucleic acid extraction step is a critical aspect of study design as this can impact the detection assays and experimental techniques that can be utilised, as well as the quality of the results and robustness of data^18,19^. The successful isolation of miRNA from small volumes of complex biological samples, such as plasma which contain high levels of RNase and contaminating proteins including albumins and globulins, is an added challenge. Commercial column-based total RNA and miRNA extraction kits are often favoured over traditional guanidium thiocyanate/phenol-chloroform RNA isolation methodologies due to reports that the latter can result in isolation of selective miRNA populations, as well as often resulting in lower quantities of impure total RNA from smaller sample volumes^14,20,21^. A similar study comparing isolation kits miRNA recovery from both plasma and cerebrospinal fluid showed that TRIzol did not perform as well as commercial extraction kits, even when combined with on-column clean-up^22^. TRIzol extracted plasma had consistently higher Cq values across multiple miRNA and lower overall RNA recovery. Several more conflicting studies over the application of the gold standard RNA isolation methods to circulating miRNA recovery have been reported, which display favourable results over kit based protocols, suggesting that there is a high degree of variability and operator influence on its success^11,23,24^. Following these reports, commercial kits optimised for biological samples such as plasma and serum which may be used with small sample volumes, are ideal for miRNA profiling and biomarker discovery in archived or clinical samples^11,25,26^.

In this study, we compared the use of six extraction kits from a range of manufacturers for their suitability for use with miRNA from either fresh or frozen ovine plasma samples. Due to the apparent variability of traditional methods in efficacy across samples and operators, we chose to examine these kits for their applicability to future miRNA profiling and potential biomarker studies. The chosen kits varied in their specificity for miRNA extraction, however, were included in the study as more broadly applicable RNA isolation kits are often cheaper and more widely used in general laboratory settings. Kits A (miRNeasy Serum/Plasma) and B (miRNeasy Serum/ Plasma Advanced) both extract total RNA, although enrich for small RNA populations <200nt. Kit C (Quick-*cf*RNA Serum and Plasma) is targeted for cell-free RNA including miRNA and mRNA, while Kit E (Isolate II) fractionates the RNA populations based on size to select for small RNAs. Both Kit E (PureLink RNA) and Kit F (Monarch Total RNA) are for total RNA populations, with no enrichment for small RNA, although they provide protocols for capturing the small RNA populations in the total RNA extract.

In addition to yield, a range of subjective operational factors were also considered, as these may influence the decision when selecting a commercial kit. Ease of use and time input were considered, as protocols must prove time-efficient across users with varying technical skill levels, especially with high-throughput experiments. The final factor of sample quantity is also of great importance for biomarker and diagnostic studies, as clinical samples are often collected in minimal volumes. Across the operational factors, the top three performing kits were the miRNeasy Serum/Plasma, miRNeasy Serum/Plasma Advanced kit, and the Quick-*cf*RNA Serum and Plasma kit.

To assess the presence of miRNA extracted when using each of the 6 kits, ten miRNA with GC content ranging from approximately 31 to 66% and varying levels of thermodynamic stability (−4.6 to 1.8 kcal/mol), were chosen for the qPCR measurement aspect of the study. While these miRNA may not cover the entire spectrum of GC content and ΔG observed in miRNA, they provide an insight into the performance of the extraction kits in contending with isolation of differing miRNA.

The miRNeasy Serum/Plasma kit was optimal across samples, displaying the least variation between fresh and frozen plasma samples as well as requiring a minimal amount of plasma input. The miRNeasy Serum/Plasma Advanced kit was the next best kit, with similar miRNA detection in fresh plasma samples and also requiring low sample input for extraction. While the Serum/Plasma Advanced kit performed worse for frozen samples, this may have been due to a lower cDNA concentration than the fresh plasma samples. The Quick-cfRNA Serum and plasma kit was the third-best kit, however showed significantly worse performance when applied to frozen plasma samples. The added requirement for extra equipment, such as a vacuum and manifold, decrease the rating on ease of use in relation to the operational factors considered as this could be an obstacle for some laboratories. The extended incubation time and sample digestion (between 1–2 hours) required by this kit also increased the time required per sample and decreased the number of samples able to be processed simultaneously.

The miRNeasy kits and the Quick-cfRNA were serum and plasma specific, suggesting that the specifically targeted kits performed better than those accommodating several sample types, such as the PureLink and Monarch Total RNA kits, and may necessitate the added cost for miRNA studies.

The PureLink RNA, Isolate, and Monarch Total RNA kits performed similarly for ovine plasma across miRNA detection and the secondary performance parameters. While the Isolate kit required the least amount of sample (100 µl), higher Cq values and lack of detection for certain miRNAs reduced the suitability of this kit for plasma miRNA studies. Issues with column clogging despite centrifugation to remove cryoprecipitates and cellular debris further contributed to the lower efficacy of this extraction kit, with similar issues noted in other challenging biological samples^27^. While the PureLink RNA and Monarch Total RNA kits involved fast and simple isolation methods, their poorer performance in detecting miRNA and greater variability between samples from fresh and frozen plasma, and across replicates of samples from the same animal, produced poorer and less reliable results. While it is worth noting that the varying elution volumes of each of the kits will impact the concentration of RNA, the reverse transcription reaction showed similar levels of cDNA and no significant difference between cDNA quantity from different extraction kits (data not shown).

One important aspect impacting the performance of extraction kits is the miRNA GC content and the stability of secondary structures^14^. While the initial report of selective loss of low GC/high ΔG miRNA populations during the extraction process was using traditional guanidium thiocyanate/phenol-chloroform reagents, it is an issue which must be investigated even in commercial kits. It is therefore of importance to assess the degree to which these kits mitigate the extraction bias of low GC/high ΔG miRNA and take this into consideration when selecting appropriate reagents.

A similar expression pattern was observed between both miRNeasy kits. While the Serum/Plasma and Serum/Plasma Advanced kits showed higher Cq values for miRNA with low GC% and high ΔG such as miR-144, −103, and −345-3p, the effect of these factors was better mitigated than in non-miRNA specific kits. Both miRNeasy kits were able to detect all miRNA and better handle the impact of low GC/high ΔG miRNA in the extraction process.

For the Quick-cfRNA kit, there were higher Cq values across all miRNA for frozen samples, however especially for miRNA with low GC% and high ΔG in frozen samples, suggesting that the freeze/thaw process may also be impacting the kits performance.

An effect of low GC/high ΔG on detection of miRNA by qPCR was also seen in the remaining kits (PureLink RNA, Isolate II, and Monarch Total RNA), however the overall higher Cq values reduced the observable magnitude and impact of these factors on general kit performance.

In comparing methods for the quantification of miRNA extracts from small volumes of ovine plasma samples, the Qubit with a microRNA assay was the least variable. The NanoDrop spectrophotometer also displayed an acceptably low level of variation between measurements, however the values provided were markedly higher than those from the Qubit and Bioanalyzer. As all measurements were taken from RNA from the same extraction and tube, we believe the high measurement values and apparent lack of sensitivity of the NanoDrop is due to the detection of contaminants such as phenol and silica carried over from extractions. Due to the dye-based methods of the other assays, these are tolerated and are not confounding measurements of nucleic acids.

Conversely, the Bioanalyzer showed high variation between sample measurements compared to the Qubit and NanoDrop, although with greater reliability in measurement values than the NanoDrop. While only two measurements per sample were taken for the Bioanalyzer compared to the 3 for the other methods, likely contributing in part to the higher variation between readings, the raw differences between the measurements can be assumed to make up much of the variation. The high cost of the Bioanalyzer chips and reagents is also disadvantage of the method, while the Qubit machine and reagents provide a more cost-effective and accessible alternative.

Thorough investigation into the ideal miRNA quantification methodologies in plasma samples have produced similar results, indicating that spectrophotometric methods may be insufficient for small quantities of plasma miRNA^15^. It appears that fluorometric assays like the Qubit may be better suited to miRNA studies, particularly when difficult samples are analysed, with our results, and others, demonstrating consistent measurement and lower variation than the microfluidic methodology of the Bioanalyzer^28^. However, only the Agilent Bioanalyzer provides a detailed quality assessment of the RNA integrity^29^. Although the operational factors showed the NanoDrop as the top method for quantification, the variability and the seemingly high measurements provided reduces its applicability to miRNA. The simple methodology of the Qubit fluorometer may also provide less room for operator error than the Bioanalyzer, which requires more technical steps including chip loading and priming and may be more applicable to multiple users and reduce time required for measurements.

All downstream steps from reverse transcription to qPCR were undertaken with the Qiagen miScript system with the same volume of RNA used for the generation of cDNA. Starting cDNA quantity did not influence amplification and qPCR performance. As this study was foremost a technical methodology assessment, levels of gene expression were not measured, and miRNA used solely to determine amplification of targets.

We have identified methodologies for the initial extraction of miRNA from plasma samples suitable for downstream use in qPCR, however further work to optimise extraction protocols for other downstream assays is of importance. The addition of carrier RNA or glycogen have been shown to increase yields and improve amplification performance^30–33^, and may be a viable option for enhanced miRNA recovery.

This study has identified suitable kits for the isolation of miRNA from ovine plasma based on a combination of qPCR performance and secondary user-focused parameters. While there was no difference between the top performing kits (miRNeasy Serum/Plasma and Serum/Plasma Advanced kits) in both qPCR performance and operational factors, the Advanced kit offers a non-toxic, phenol-free alternative for miRNA extraction. This allows the protocol to be undertaken on the bench without the need for specialist protective equipment and reduces the time required per sample, by replacing the phase separation step. Studies using human plasma have found similar results regarding these kits and their favourable performance in the isolation of miRNA suitable for qPCR detection assays, supporting the conclusion that column-based extraction methods are optimal for complex biological samples^31,34,35^. Overall, the miRNeasy Serum/Plasma and Serum/Plasma Advanced kit displayed superior qPCR performance, however as the Advanced kit and did not require long incubation and digestion times it was considered to be the most applicable kit. When used with the Qubit microRNA assay, concentrated and pure miRNA can be extracted from small volumes of either fresh or frozen plasma and measured with a high degree of sensitivity.

## Methods

### Plasma collection

Whole blood was collected into EDTA-coated vacutainer tubes (BD, Australia) via jugular venepuncture from 5 healthy sheep. All samples were processed on the day of collection and plasma separated by centrifugation at 2,000 × *g* for 20 minutes at 4 °C (Beckman Coulter Allegra X-12R). The supernatant was then transferred to a sterile tube leaving approximately 1 cm above the buffy coat to avoid contamination with white blood cells and platelets. Plasma was aliquoted into 200 µl volumes, visually inspected for haemolysis, and either frozen at −80 °C or stored at 4 °C for immediate RNA extraction.

### RNA extraction

RNA was extracted from either fresh or frozen plasma according to the manufacturer’s instructions using 6 commercially available kits; Kit A: miRNeasy Serum/Plasma (Qiagen, Germany), Kit B: miRNeasy Serum/Plasma Advanced (Qiagen, Germany), Kit C: Quick-*cf*RNA Serum & Plasma (Zymo Research, USA), Kit D: Isolate II miRNA (Bioline, Australia), Kit E: PureLink RNA Mini kit (Invitrogen, USA), Kit F: Monarch Total RNA Miniprep kit (New England BioLabs, USA). All extractions were performed in duplicate for each animal, except for the PureLink and Monarch kits due to sample and reagent availability. Following extraction and elution of RNA, samples were immediately frozen at −80 °C.

#### Kit A: miRNeasy Serum/Plasma kit

Briefly, Total RNA was isolated from 200 µl of plasma as per the manufacturer’s recommendations and eluted in 14 µl of RNase-free water.

#### Kit B: miRNeasy Serum/Plasma Advanced kit

The miRNeasy Serum/Plasma Advanced kit is a phenol-free alternative to the original miRNeasy Serum/Plasma kit. Total RNA was extracted from 200 µl of plasma according to the manufacturer’s protocol and eluted in 20 µl of RNase-free water.

#### Kit C: Quick-cfRNA Serum & Plasma kit

Total RNA was extracted from 200 µl of plasma according to the manufacturer’s instructions using a vacuum pump and manifold for initial sample processing. RNA was eluted in 15 µl of RNase-free water.

#### Kit D: Isolate II miRNA kit

A 100 µl aliquot of plasma was used for extractions with the Isolate II kit, as per manufacturer’s instructions. Enriched small RNA (<200nt) was eluted in 50 µl of RNA Elution Buffer, the minimum amount required to adequately wet the column.

#### Kit E: PureLink RNA mini kit

Total RNA was isolated from 200 µl of plasma as per the manufacturer’s recommendations and RNA eluted in 30 µl of RNase-free water.

#### Kit F: Monarch total RNA miniprep kit

As per the manufacturer’s recommendations, total RNA was extracted from 200 µl of plasma and eluted in the minimum suggested volume of 50 µl of RNase-free water.

### Comparison of RNA quantification methods

Three miRNA quantification methods, NanoDrop, Qubit Fluorometer, and Agilent 2100 Bioanalyzer, were compared for their suitability to provide a reasonable measurement of RNA quantity extracted from plasma. RNA was extracted from 200 µl of frozen plasma using the Qiagen miRNeasy Serum/Plasma Advanced kit and frozen at −80 °C. A single kit was used to avoid variability arising from isolation method, and the Serum/Plasma Advanced kit chosen due to the low time input required and the phenol-free formula. All measurements were performed in triplicate, except for the Bioanalyzer, from 5 biological replicates. Measurements were taken from the same RNA eluate and mixed via pipetting prior to sample loading.

#### NanoDrop spectrophotometer

A NanoDrop One UV-Vis spectrophotometer (ThermoScientific) was used for RNA assessment this utilises the absorbance of light by nucleic acids to determine their concentration. Prior to measurement, the NanoDrop was blanked with 2 µl of the same nuclease-free water that the RNA was eluted in. Briefly, 2 µl of each RNA sample was loaded onto the NanoDrop pedestal and the arm lowered. Absorbance measurements were taken for each sample and the concentration (ng/µl) calculated.

#### Qubit® microRNA assay

The Qubit® microRNA Assay Kit was used alongside the Qubit® 2.0 Fluorometer (Life Technologies, USA) to measure the quantity of RNA in each sample. With a stated core detection range of 5–500 ng/mL, the microRNA Assay Kit uses fluorometric detection of a miRNA specific dye to determine concentration, and provides 2 standards, 0 ng/µl and 10 ng/µl, to generate a standard curve prior to sample measurement. Each standard (10 µl) was diluted in 190 µl of working solution (1:200 dilution of microRNA Reagent in microRNA Buffer), while 2 µl of RNA sample was diluted in 198 µl of working solution. The mixtures were vortexed and incubated for 2 minutes in the dark at room temperature before measurements were taken. Qubit values were provided as ng/mL, and sample concentration was calculated using the Qubit® 2.0 Fluorometer’s dilution calculator.

#### Agilent 2100 bioanalyzer

Evaluation of RNA quantity was assessed using the Agilent Bioanalyzer and Small RNA chip and reagents. This system employs microfluidic technology to electrophoretically separate and fluorescently label nucleic acids including RNA and miRNA. Small RNA chips and reagents were primed and prepared according to the manufacturer’s instructions. Briefly, 2 µl of dye was mixed with 40 µl of filtered gel mixture and centrifuged at 13,000 *x g* for 10 minutes. Gel-dye mix (9 µl) was pipetted into the appropriate well and the chip primed, followed by 9 µl of gel-dye mixture into the remaining gel wells. Conditioning solution (9 µl) was added to appropriate well, and 5 µl of the Small RNA marker to each sample and ladder well. The heat denatured ladder (70 °C for 2 minutes) was pipetted into the ladder well, and 1 µl of each denatured sample into each of the sample wells. The chip was then vortexed for 60 seconds and read using the Agilent 2100 Bioanalyzer.

### Reverse transcription and cDNA synthesis

Reverse transcription for all RNA extracts was performed using the miScript RT kit (Qiagen, Germany) according to the manufacturer’s instructions. A master mix was prepared on ice containing 4 µl of 5x HiSpec Buffer, 2 µl of 10x Nucleics Mix, 2 µl of Reverse Transcriptase, and 2 µl of RNase-free water; per reaction to a total volume of 20 µl. 10 µl of master mix was added to 10 µl of the total RNA extracted from each plasma sample (n = 100) and incubated in a Corbett PalmCycler 96 thermal cycler using the following conditions: 37 °C for 60 minutes, followed by 95 °C for 5 minutes to inactivate the reverse transcriptase. cDNA was kept on ice and diluted with 200 µl of RNase-free water. Diluted cDNA was stored at −80 °C until use.

### Quantification of cDNA

cDNA synthesis efficiency and success were determined using a Qubit® ssDNA assay and the Qubit® 1.0 fluorometer. Two standards provided with the kit (0 ng/µL and 20 ng/µL) were made from the stock solutions and used to generate a standard curve. Briefly, 10 µl of each standard was diluted in 190 µl of working solution (1:200 dilution of ssDNA Reagent in ssDNA buffer), while 2 µl of sample was diluted in 198 µl of working solution. The mixtures were vortexed and incubated for 2 minutes in the dark at room temperature before measurements were taken. Measurements were used to estimate starting concentration of the RNA sample, qPCR cDNA input, and the efficiency of the reverse transcription reaction from a consistent input volume of 10 µL.

### qPCR

Quantitative (q)PCRs were carried out using an Mx3000p Real-time PCR system (Stratagene, Agilent) and the miScript SYBR Green PCR Kit (Qiagen, Germany) for 10 miRNA with varying GC content and thermostability (ΔG, kcal/mol) (Supplementary Figure 2). miScript mature miRNA primers were obtained from Qiagen and reconstituted in 550 µl of TE (pH 8.0) prior to use. Assays set up in 96-well PCR plates (Integrated Sciences, Australia) included duplicates of each individual sample, No Template Controls (NTC) and No Reverse Transcriptase Controls (NRT). As there were 2 extractions for each animal and 2 qPCR duplicates, there were a total of 4 replicates per animal (with the exception of Kits E and F as previously stated having 2 replicates).

Total reaction volumes were 25 µl, including 5 µl of cDNA, 12.5 µl of QuantiTect SYBR Green Master Mix (Qiagen, Germany), 2.5 µl of each of the miScript specific primer and the miScript Universal Primer, and 2.5 µl of nuclease-free water. Amplification was under the following conditions: 95 °C for 15 minutes; 40 cycles of 94 °C for 15 seconds, 55 °C for 30 seconds, 70 °C for 30 seconds with fluorescence data acquisition occurring at the end of each cycle, followed by 1 cycle of 95 °C for 1 minute, 65 °C for 30 seconds, and 97 °C for 30 seconds. Cq values represent the cycle at which the detected fluorescence crosses the threshold. A dissociation curve was generated with ovine samples run alongside a bovine control to ensure amplification of the correct PCR product and homology of miRNA sequences across both species. Any samples with no amplification values were re-tested to confirm the result.

### Statistical analysis

Raw Cq values were transformed to efficiency-weighted Cq (Cw) values prior to analysis using the common base method^36^. This method accounts for each technical replicate, as well as the efficiency of the reaction to control for differences between qPCR runs and plate to plate variability and transforms raw Cq values to a normal logscale distribution.

Statistical analyses were performed using Genstat (v18.0). Data were grouped according to treatment (i.e. fresh or frozen plasma), gene, and extraction kit. A REML (restricted maximum likelihood) with Fishers LSD was performed to measure significant differences between the means of different treatments and kits, and accounting for the varying GC content of each miRNA.

A two-way ANOVA was used to analyse the cDNA quantity for fresh and frozen samples and assess variation in the cDNA input into the PCR reaction for both fresh and frozen samples. The interaction between kit and treatment was evaluated and significance assessed at the 5% level.

For each of the quantification methods, the coefficient of variation (CV) was calculated for intra-measurement variation and expressed as a percentage, as well as the standard error of the mean (SEM).

### Ethics approval and consent to participate

All procedures were carried out under approval from the University of Sydney’s Animal Ethics Committee (AEC No. 2017/2149).

## Supplementary information

Supplementary figures.

## Data Availability

Analysed data are presented within the current manuscript (Tables 1 and 2 and Figs. 1–5). Individual raw data is available from the corresponding author upon reasonable request.
