# Supplementary material for: Comparison of methods for miRNA isolation and quantification from ovine plasma
Source: Sci Rep. 2020 Jan 21;10:825. doi: 10.1038/s41598-020-57659-7 (PMC6972740; doi:10.1038/s41598-020-57659-7)
Supplement: Supplementary file 1 — Supplementary figures. [file 41598_2020_57659_MOESM1_ESM.pdf]

Supplementary information for:

Comparison of methods for miRNA isolation and quantification from ovine plasma

AUTHORS: Kathryn Wright[1], Kumudika de Silva[1]*, Auriol Purdie[1], Karren Plain[1]

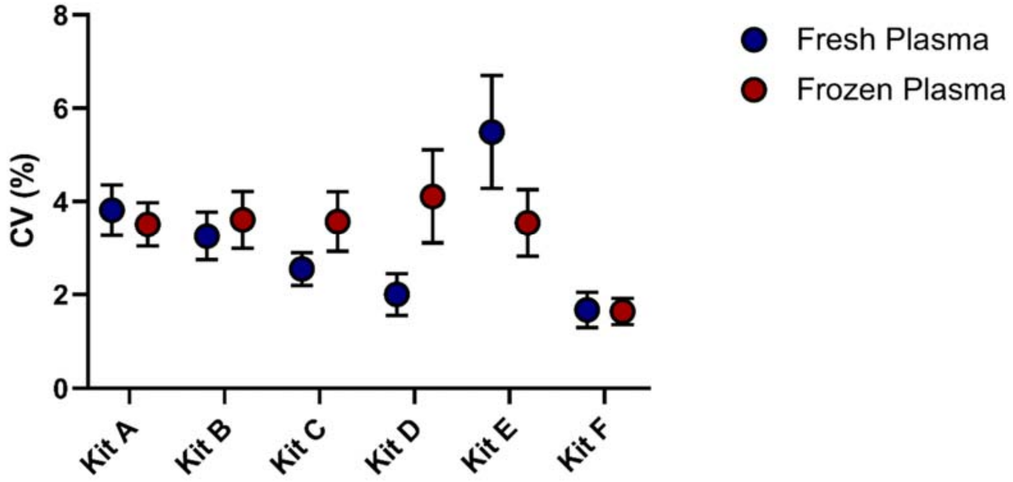

Supplementary Figure 1. Variability of technical replicates from fresh and frozen plasma samples.

For each kit, the median coefficient of variation (CV) and SEM across all miRNA was calculated from Cq values. For kits A-C & E, 2 extraction replicates and 2 qPCR replicates were performed for 5 biological replicates across 10 miRNA. For Kits D & F, 1 extraction and 2 qPCR replicates were performed for the 5 biological replicates and 10 miRNA.

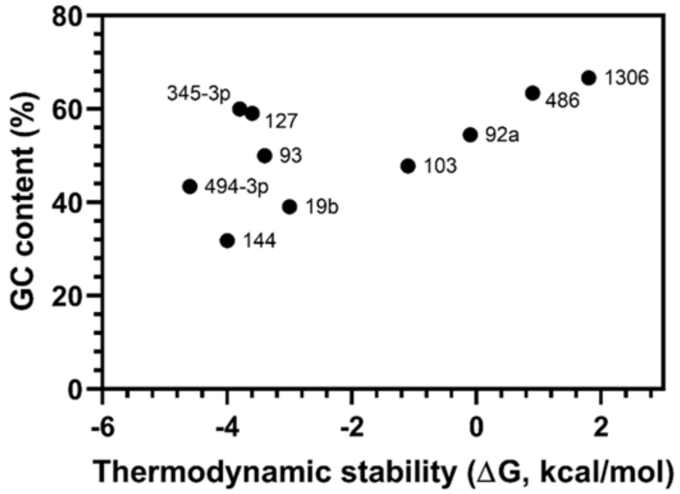

Supplementary Figure 2. GC and thermostability range of selected miRNA

The miRNA selected for profiling in this study to assess the effect of low GC content and secondary structure stability. GC content (%) is plotted against the thermodynamic stability, determined using Mfold.
